# Response: Commentary: Cognitive behavioural therapy for the treatment of chronic fatigue syndrome in adults: a short analysis of the meta-analysis

**DOI:** 10.3389/fpsyt.2026.1818125

**Published:** 2026-04-30

**Authors:** Vivek Kolala, Billie La Rosa, Venkat Vangaveti, Kai Yang Chen

**Affiliations:** 1 Canberra Health Services, Canberra, ACT, Australia; 2 Queensland Health, Townsville, QLD, Australia; 3 James Cook University, Townsville, QLD, Australia

**Keywords:** chronic fatigue syndrome, cognitive behavioural therapy, fatigue, meta-analysis, *Myalgic encephalomyelitis*, physical functioning, psychological treatment

## Discussion

We thank Vink et al. for their critique of our meta-analysis of Cognitive Behavioural Therapy (CBT) for the treatment of chronic fatigue syndrome (CFS) in adults (1). The conclusions of our publication were that individual face-to-face CBT showed efficacy in reducing fatigue, while self-directed CBT showed efficacy in improving physical functioning. No serious adverse effects were reported, although they were variably documented in included randomised controlled trials (RCTs). These findings should be interpreted cautiously given the high heterogeneity across studies. The authors recommended further research into the role of specific CBT modalities in CFS (2). We provide clarifications and responses to the primary concerns raised by Vink et al. below.

Vink et al. suggest that analyses by CBT delivery modality represents an undisclosed *post-hoc* deviation from protocol. While subgroup analyses by CBT modality were not explicitly specified in the original PROSPERO registration, they were conducted as exploratory sensitivity analyses and were clearly described in the published Methods (2). These analyses did not alter eligibility criteria, outcomes, or study selection, and were interpreted cautiously. Furthermore, this was performed in addition to investigating the efficacy of all CBT modalities pooled together.

The authors further state that our review was designed to examine “non-protocol-based CBT” and that trials using protocolised or time-contingent CBT should therefore have been excluded. This appears to reflect a misunderstanding, as our protocol was not set out to exclude non-protocol based CBT interventions.

Vink et al. argue that Prins et al. and Deale et al. should have been excluded on the basis that they used the Oxford criteria (3, 4). Our exclusion criteria applied only to studies that explicitly and exclusively used the Oxford criteria, which Prins et al. and Deale et al. did not. Our exclusion of Oxford-defined cohorts was not based solely on the absence of mandatory post-exertional malaise as Vink et al. suggest, but on the broader qualitative distinction that the Oxford criteria define CFS primarily as a fatigue-dominant condition rather than a syndromic illness, and have been specifically criticised and discouraged by expert bodies (5, 6).

Vink et al. argue that several included trials should have been rated as high risk of bias due to refusal rates, non-blinding, reliance on patient-reported outcomes and non-adherence. Under the Cochrane Risk of Bias 2 framework, these issues map to distinct domains and do not necessarily mandate automatic high-risk judgements (7). Participant refusal prior to randomisation and sample representativeness relate to external validity and fall outside the scope of RoB2 (8). Furthermore, interpreting refusal as evidence of non-improvement or harm is speculative and extends beyond what the available data can support (9).

With respect to non-blinding, Vink et al. also add that several included trials used waiting-list or natural course comparators rather than active controls, and that inactive comparators may inflate effect estimates in non-blinded studies. We acknowledge this as a recognised limitation of the underlying trial designs, and have commented on the difficulty with blinding in psychological trials. Our inclusion criteria specified non-CBT comparators, encompassing a range of control conditions, of which waiting-list controls represented one but not the only comparator type. These comparator limitations are therefore best understood as constraints of the primary evidence base and were handled through cautious interpretation rather than exclusion of otherwise eligible trials. Rob2 requires reviewers to assess whether outcome measurement is likely to have been meaningfully influenced by knowledge of the intervention, which involves judgement rather than a prescriptive rule. We applied RoB2 in accordance with Cochrane guidance and did not assume high risk solely based on non-blinding (7).

Regarding patient reported outcomes, as noted in the Cochrane Handbook, “PROs (patient reported outcomes) are essential when externally observable patient-important outcomes are rare or unavailable … including … fatigue.” Appropriate response is cautious interpretation and triangulation, rather than exclusion of patient-reported measures (10). Not all clinically meaningful outcomes can be measured objectively, especially in conditions such as CFS where subjective symptom experience is central.

Attrition and non-adherence are considered under bias due to deviations from intended interventions and missing outcome data, where judgements depend on evidence of differential, outcome-related deviations and the analytic approach used, rather than numerical thresholds such as the 5 and 20 rule proposed by Vink et al. RoB2 does not endorse fixed cut-offs and instead requires domain-specific, contextual judgement guided by signalling questions (8). We applied RoB2 in accordance with this guidance, and our conclusions were interpreted cautiously in recognition of these limitations. For example, in Janse et al., Vink et al. cite “81–84% non-adherence” figure is derived from a strict “full adherence” definition that differed between arms (e.g., the protocol-driven condition required ≥12 emails in addition to opening modules) and was sensitive to whether the relapse module was counted; when the relapse module was excluded, adherence was substantially higher (e.g., 49% and 93% by arm) (11). Accordingly, this was considered under RoB2 Domain 2 as having some concerns rather than treated as automatic grounds for a high-risk judgement (12).

Vink et al. highlight that absolute post-treatment fatigue and disability scores remain elevated in several trials. Our review did not claim that CBT leads to cure, normalization of functioning, or resolution of disability.

Vink et al. state that the included self-directed CBT trials did not require impaired physical functioning at baseline, which limits interpretation of clinical significance. However, absence of an entry requirement does not preclude assessment of change in physical functioning or warrant exclusion. Our review does not equate statistical effects with normalization or recovery.

We also acknowledge that the self-directed CBT trials originated from a single centre, which may limit generalisability. This does not preclude their inclusion in a systematic review, but underscores the need for replication with further trials, which is a recommendation we have outlined in the article. Research funding and trial infrastructure are often concentrated within specialist academic centres with the resources required to conduct complex interventions, however future multi-centre replication is recommended (13, 14).

Vink et al. critiqued our handling of adverse effect reporting. In the meta-analysis, harms data were pooled across trials to provide a collective assessment. While reporting was inconsistent, no serious adverse events were identified within trial definitions. Findings from Núñez et al. were included within this synthesis (15). We have emphasised the need for improved adverse event reporting in future studies.

In conclusion, we respectfully disagree with Vink et al.’s critique. Our meta-analysis adhered to prespecified eligibility criteria and applied the Cochrane Risk of Bias 2 framework as intended, with domain-specific judgements and cautious interpretation rather than automatic exclusion or high-risk classification. Our conclusions do not equate statistical effects with recovery and explicitly acknowledge limitations within the existing evidence base.
